# Targeted next generation sequencing identifies novel NOTCH3 gene mutations in CADASIL diagnostics patients

**DOI:** 10.1186/s40246-016-0093-z

**Published:** 2016-11-24

**Authors:** Neven Maksemous, Robert A. Smith, Larisa M. Haupt, Lyn R. Griffiths

**Affiliations:** Genomics Research Centre, Institute of Health and Biomedical Innovation (IHBI), School of Biomedical Sciences, Queensland University of Technology (QUT), Q Block, 60 Musk Ave, Kelvin Grove Campus, Brisbane, 4059 Queensland Australia

**Keywords:** AmpliSeq Custom Panel, CADASIL, Next-generation sequencing, *NOTCH3*

## Abstract

**Background:**

Cerebral autosomal dominant arteriopathy with subcortical infarcts and leukoencephalopathy (CADASIL) is a monogenic, hereditary, small vessel disease of the brain causing stroke and vascular dementia in adults. CADASIL has previously been shown to be caused by varying mutations in the *NOTCH3* gene. The disorder is often misdiagnosed due to its significant clinical heterogeneic manifestation with familial hemiplegic migraine and several ataxia disorders as well as the location of the currently identified causative mutations. The aim of this study was to develop a new, comprehensive and efficient single assay strategy for complete molecular diagnosis of *NOTCH3* mutations through the use of a custom next-generation sequencing (NGS) panel for improved routine clinical molecular diagnostic testing.

**Results:**

Our custom NGS panel identified nine genetic variants in *NOTCH3* (p.D139V, p.C183R, p.R332C, p.Y465C, p.C597W, p.R607H, p.E813E, p.C977G and p.Y1106C). Six mutations were stereotypical CADASIL mutations leading to an odd number of cysteine residues in one of the 34 *NOTCH3* gene epidermal growth factor (EGF)-like repeats, including three new typical cysteine mutations identified in exon 11 (p.C597W; c.1791C>G); exon 18 (p.C977G; c.2929T>G) and exon 20 (p.Y1106C; c.3317A>G). Interestingly, a novel missense mutation in the *CACNA1A* gene was also identified in one CADASIL patient. All variants identified (novel and known) were further investigated using in silico bioinformatic analyses and confirmed through Sanger sequencing.

**Conclusions:**

NGS provides an improved and effective methodology for the diagnosis of CADASIL. The NGS approach reduced time and cost for comprehensive genetic diagnosis, placing genetic diagnostic testing within reach of more patients.

**Electronic supplementary material:**

The online version of this article (doi:10.1186/s40246-016-0093-z) contains supplementary material, which is available to authorized users.

## Background

The stroke syndrome CADASIL [MIM 125310] (cerebral autosomal dominant arteriopathy with subcortical infarcts and leukoencephalopathy) disorder results in neuronal white matter abnormalities and is characterised by a variety of symptoms including, vascular degeneration, recurrent subcortical ischaemic strokes, progressive cognitive decline, dementia, migraine with aura (22 % of patients) and premature death [1]. The unique deposition of granular osmiophilic material (GOM) in systemic and brain vasculature differentiates CADASIL patients from those suffering similar hereditary vascular disorders [2]. CADASIL is often misdiagnosed due to its significant clinically heterogeneic manifestation with familial hemiplegic migraine and several ataxia disorders, as these disorders have an autosomal dominant mode of inheritance and share clinical characteristics such as hemiplegic migraine, migraine with typical aura and progressive ataxia [3–6]. Mutations implicated in CADASIL have been identified on chromosome 19, specifically within *NOTCH3* (MIM 600276), which encodes a transmembrane receptor primarily expressed in vascular smooth muscle cells. *NOTCH3* located at 19p13 is 33 exons long and spans approximately 7 kb [4]. Currently, at least 200 mutations resulting in an odd number of cysteine residues are known to be associated with CADASIL. These mutations all occur in exons 2–24 of *NOTCH3* that encode 34 epidermal growth factor (EGF)-like repeats in the extracellular domain of the NOTCH3 protein. The large number of exons combined with their high GC content makes comprehensive sequencing of this gene with traditional Sanger sequencing (SS) expensive and time consuming. With the advent of next-generation sequencing (NGS), the sequencing of target genes, regions, exomes or whole genomes, provides cost-effective, high-throughput screening suitable for molecular diagnostics enabling detection of a wide array of mutations with sensitivity and specificity. Here, we have performed targeted gene sequencing using a custom five-gene NGS panel [7], encompassing the coding sequences, 20–100 bp exon/intron boundaries and the 5′ and 3′UTR regions of *NOTCH3* in 44 patients.

## Results

### NGS-panel sequencing output

The sequencing output data from the Ion Torrent PGM was analysed using the Ion Torrent platform-specific software Torrent Suite V3.6 (Thermo Fisher Scientific, Scoresby, Victoria, Australia). The 44 samples were sequenced using seven different Ion 316 chips, to generate an average sequencing of 3,303,300 total reads, 477.4 Mb total bases sequenced, and 472.2 Mb with 99 % of bases aligned to the human complete genome (hg19) per Ion 316 chip. For all samples sequenced, the average read depth across the target region was 560.65×, while the average percentage of target bases covered at 20× or greater was 96 % and the average uniformity of coverage was 90.64 %.

### Sequencing data analysis

Comprehensive screening for *NOTCH3* using the AmpliSeq Custom NGS panel [7] (Thermo Fisher Scientific, Scoresby, Victoria, Australia) for targeted gene sequencing was conducted on 44 patients, previously screened for standard sequencing exons (3 and 4) and/or (2,11, 18 and 19) by SS and classified as being negative for known mutations.

Initial analysis using the IonReporter software (Thermo Fisher Scientific, Scoresby, Victoria, Australia) identified 42 variants scattered over *NOTCH3* among the 44 patients. An overview of all variants detected in our study is shown in Additional file 1: Table S1 online. Of these, nine particularly notable genetic variants were identified in 10 patients (22.7 %) out of the 44 subjects: five novel potential mutations (NOTCH3:NM_000435:exon4:c.416A>T:p.D139V, exon1 1:c.1791C>G:p.C597W and c.1820G>A: p.R607H, exon18: c.2929T>G,: p.C977G; and exon20: c.3317A>G: p.Y1106C); three previously reported disease-causing missense mutations (NOTCH3:NM_000435:exon4:c.547T>Cp.C183R [8], exon6:c.994C>T, p.R332C [9, 10]; and exon9: c.1394A>G: p.Y465R [11]) and one novel synonymous genetic variant in NOTCH3:NM_000435:exon16:c.2439G>A: p.E813E [Tables 1 and 2]. Clinical information for all samples was not available [see Additional file 2: Table S2]; however, the following clinical parameters were attributed to the relevant samples in our cohort: (i) white matter abnormalities were seen in patients with E813E, C183R and R332C mutations; (ii) positive skin biopsy signs were reported in patients with C977G, Y1106C, Y465C and C183R and (iii) a family history of dementia and/or stroke was reported in patients with E813E, C977G, Y1106C, C183R, R332C and R465C mutations.

**Table 1:** Variants of unknown significance of *NOTCH3*, *CACNA1A* and *SCN1A* genes identified in eight suspicious CADASIL patients. RefSeq NM_000435, NM_001127221 and NM_001165963

Sample ID	Gene	Gender	Age	Exon	EGF-repeat	Codon change (FWD)	Protein change	PhyloP	SIFT	PolyPhen2 HVar	MutationTaster	GERP++	AGVGD	PhD-SNP
C-11	NOTCH3	F	42	4	3	c.416A>T	p.Asp139Val	C (1.89)	T (0.06)	P (0.499)	D	5.02	C65	Non-neutral
C-4	NOTCH3	M	67	11	15	c.1791C>G	p.Cys597Trp	N (0.434)	D (0)	D (1.0)	D	2.25	C65	Non-neutral
C-15	NOTCH3	F	52	11	15	c.1820G>A	p.Arg607His	C (2.21)	T (0.54)	B (0.0.026)	D	3.22	C25	Neutral
C-24	NOTCH3	M	54	16	21	c.2439G>A	p.Glu813Glu	–	–	–	D (splice site changes)	–	–	–
C-10 and C-44	NOTCH3	F	74, 52	18	25	c.2929T>G	p.Cys977Gly	C (2.04)	D (0)	D (1.0)	D	5.36	C65	Non-neutral
C-6	NOTCH3	F	51	20	28	c.3317A>G	p.Tyr1106Cys	C (1.92)	D (0)	D (0.998)	D	5.08	C65	Non-neutral
C-36	CACNA1A	M	60	6	–	c.832G>T	p.Ala278Ser	C (2.46)	T (0.13)	D (0.963)	D	5.27	NA	Neutral
C-36	SCN1A	M	60	20	–	c.3924A>T	p.Glu1308Asp	C (2.08)	T (0.37)	P (0.727)	D	5.46	NA	Neutral

PhyloP, SIFT, Polyphen-2, MutationTaster, GERP++, AGVGD and PhD-SNP are functional prediction scores in which increasing values indicate a more damaging effect except SIFT score <0.05 has damaging effect

*Abbreviations*: *C* conserved, *N* not-conserved or neutral, *D* damaging or deleterious, *P* possible damaging, *T* tolerated, *B* benign, *NA* not applicable

**Table 2:** Variants of known *NOTCH3* mutations identified in three patients by NGS. RefSeq NM_000435.2

Sample ID	Gender	Age	Exon	EGF-repeat	Codon change	Protein change	PhyloP	SIFT	PolyPhen2 HVar	MutationTaster	GERP++	AGVGD	PhD-SNP	Snp138
C-20	F	54	4	4	c.547T>C	p.Cys183Arg	C (1.82)	D (0)	D (1)	D	4.32	C65	Non-neutral	
C-42	F	47	6	8	c.994C>T	p.Arg332Cys	C (2.46)	D (0.03)	D (1)	D	4.6	C65	Non-neutral	rs137852641
C-14	M	33	9	11	c.1394A>G	p.Tyr465Cys	N (−0.833)	T (0.08)	P (0.886)	D	−4.41	C65	Non-neutral	

PhyloP, SIFT, Polyphen-2, MutationTaster, GERP++, AGVGD and PhD-SNP are functional prediction scores in which increasing values indicate a more damaging effect except SIFT score <0.05 has damaging effect

*Abbreviations*: *C* conserved, *N* not-conserved or neutral, *D* damaging or deleterious, *P* possible damaging, *T* tolerated

Molecular genetic testing using the custom NGS panel encompassing five genes (*NOTCH3*, *CACNA1A*, *ATP1A2*, *SCN1A* and *TRESK* genes) identified two remarkable variants in case C-36 (CACNA1A:NM_023035:c.832G>T:p.A278S and SCN1A:NM_006920:c.3924A>T: p.E1308D [Table 1]). These mutations correspond to highly conserved amino acid residues according to four in silico prediction tools (PhyloP of score of >2.0, PolyPhen2 HVar of score >0.7, and MutationTaster with a damaging effect and GERP++ score above 5).

In addition to these variants, nine rare single nucleotide polymorphisms (SNPs) in the *NOTCH3* gene with minor allele frequency (MAF) ≤0.1 % were observed in nine patients with no other causative mutation found in *NOTCH3* [Additional file 1: Table S1 online and Table 3]. One patient (case C-3) was shown to carry two rare amino acid changing variants p.S497L and p.A1020P in exons 9 and 19 of *NOTCH3*, respectively. All nine SNPs were further assessed by seven in silico prediction programmes with three of these variants (p.S497L, p.P496L and p.Y220Y) shown to have a damaging effect by MutationTaster.

**Table 3:** Rare variants identified by NGS in the *NOTCH3* gene. RefSeq NM_000435.2

Patient ID	Locus.	Ref	Location	Codon change	Protein change	PhyloP	SIFT	PolyPhen	LRT	MutationTaster	GERP++	AGVGD	PhD-SNP	dbSNP	MAF
C31	chr19:15281342	T	Ex27	c.4914A>G	WT (p.Glu1638Glu)	0.25				Poly				rs149222385	0.001
C13, C28, C33	chr19:15290236	G	Ex21	c.3399C>A	p.His1133Gln	−2.55	D	P (0.68)	NA	Poly	−8.6	C15	Neutral	rs112197217	0.005
C3, C12	chr19:15291576	C	Ex19	c.3058G>C	p.Ala1020Pro	0.57	T (0.19)	B (0.054)	N (0.006)	Poly	1.44	C25	Neutral	rs35769976	0.083
C3	chr19:15296513	C	In12			0.5				Poly				rs147014533	0.006
C3	chr19:15299048	G	Ex9	c.1490C>T	p.Ser497Leu	2.35	T (0.33)	B (0.036)	N (0.018)	D	5.04	C65	Neutral	rs114207045	0.006
C34	chr19:15299051	G	Ex9	c.1487C>T	p.Pro496Leu	2.35	T (0.1)	p (0.883)	N (0.007)	D	5.04	C65	Disease	rs11670799	0.005
C9	chr19:15302790	G	Ex4	c.660C>T	WT (p.Tyr220Tyr)	−1.85				D				rs114457076	0.001
C3	chr19:15308287	G	In2			−0.16				Poly				rs188132716	0.006
C16	chr19:15308288	G	In2			−0.98				Poly				rs202151374	0.003

PhyloP, SIFT, Polyphen-2, Mutation Taster, GERP++, AGVGD, and PhD-SNP are functional prediction scores in which increasing values indicate a more damaging effect except SIFT score <0.05 has damaging effect

*Abbreviations*: *B* benign, *C* conserved, *D* damaging or deleterious, *Ex* exon, *In* intron, *NA* not applicable, *N* not-conserved or neutral, *P* possible damaging, *Poly* polymorphic, *T* tolerated, *WT* wild type

**Fig. 1:**
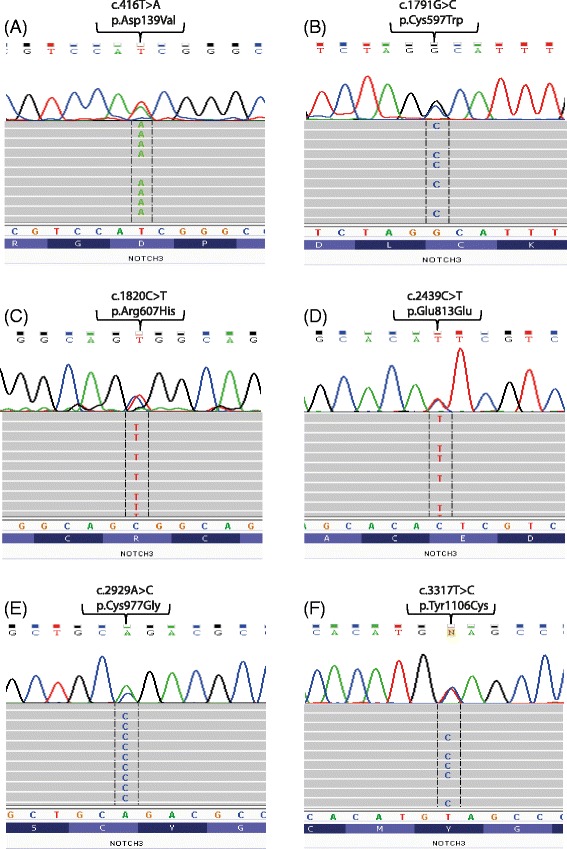
Sequences (reverse complement) of the six novel genetic variants in *NOTCH3* identified by NGS. The figure shows the six heterozygous exonic point variants: **a** p.D139V in exon 4, **b** p.C597W in exon 11, **c** p.R607H in exon 11, **d** synonymous variant p.E813E in exon 16, **e** p.C977G in exon 18 and **f** p.Y1106C in exon 20 identified in this study. Only bases non-concordant with consensus sequence are displayed in the target reads with the integrative genomics viewer IGV [12]. The normal nucleotide and protein sequences are depicted at the *bottom* and *top* of the figure

All variants detected by NGS and reported in this study were visually confirmed using Integrative Genomics Viewer (IGV v2.3) software [12] and compared with NCBI reference sequences [13]. In order to verify the accuracy of potential novel mutations identified by NGS, SS was performed for all samples with the five non-synonymous variants along with the synonymous new variant showing complete consistency (100 %) between the two methods [Fig. 1].

Analyses of the potential functional significance of the six novel *NOTCH3* genetic variants identified the C597W, C977G and Y1106C missense mutations to be pathogenic by six of the seven genetic prediction software programmes (PhyloP, SIFT, PolyPhen2, MutationTaster, AGVGD and PhD-SNP) [Table 1].

Finally, we compared the potential functional significance of the three known pathogenic missense mutations (C183R, R332C and Y465C) with the six novel genomic variants identified using the same seven in silico software programmes. The C183R and R332C mutations showed a high potential damaging effect when analysed by all seven programmes used; in contrast, the Y465C mutation showed a tolerated or benign effect in four of the seven in silico programmes [Table 2].

## Discussion

Molecular genetic testing is an essential tool for accurate CADASIL diagnosis. Several diagnostic approaches have been used for CADASIL, in particular the use of skin biopsy to detect unusual NOTCH3 expression. However, despite the widespread use of biopsy testing, the low sensitivity of this method in CADASIL diagnosis has been reported [14]. In addition, previous work by Markus et al. tested the sensitivity of single strand conformation polymorphism (SSCP) analysis for detecting *NOTCH3* mutations, with an effective success rate of 80 to 85 % [14]. More recently, He et al. reported that varying and population-dependant results in the effectiveness of using the pre-genetic “CADASIL scale” screening tool which evaluates clinical presentations and neuroimaging data in an effort to minimise *NOTCH3* gene testing [15, 16]. As such, current diagnosis relies on the screening of all exons by sequencing to identify mutations in *NOTCH3*.

We have previously demonstrated the efficiency of our NGS panel for detecting known and novel mutations in a cohort of episodic ataxia patients and increasing the rate of mutation detection by 48 % [7]. We have now utilised this custom targeted massively parallel NGS panel to examine the coding sequences, intron/exon boundaries including 20–100 bases of flanking intronic nucleotides and the 5′ and 3′UTR regions of *NOTCH3* in a cohort of 44 patients with clinically suspicious CADASIL.

Targeted gene sequencing analysis efficiently identified nine novel genetic variants in *NOTCH3*, of which five non-synonymous mutations (p.D139V, p.C597W, p.R607H, p.C977G and p.Y1106C) and one synonymous variant (p.E813E) have not been previously described. In addition, three missense mutations previously reported as pathogenic (C183R [8], R332C [9, 10] and Y465C [11]) but not previously identified in our diagnostics cohort were also detected [17]. In total, six typical CADASIL mutations involving cysteine alterations were identified in seven patients (15.9 %) out of 44 subjects, a detection rate higher than previously reported by Fernandez et al. and Bianchi et al. [18, 19].

Interestingly, previous studies have revealed differences in the spectrum of *NOTCH3* mutations between Asian and Italian populations and populations of Caucasian ethnicity [18, 20, 21]. Our results also showed no evidence of strong clustering of *NOTCH3* mutations in specific exons. The variants identified in this study occur in seven different exons (4, 6, 9, 11, 16, 18 and 20) within the EGF-like repeat regions of the gene. The patient cohort encompasses different ethnic backgrounds, reflecting the diversity of the Australian population. This highlights the potential confounding factor in nations of multiple ethnic backgrounds, where mutations may occur at multiple sites making molecular diagnosis difficult and time consuming if using traditional SS methodologies. While exonic clustering in ethnic groups is likely due to founder effects, de novo mutations resulting in mutations in ethnically homogenous populations are possible. In this instance, the use of SS may still miss mutations in a proportion of patients suggesting that screening of all coding regions in *NOTCH3* is of benefit for the comprehensive molecular diagnosis of CADASIL.

Six of the missense mutations identified were stereotypical CADASIL mutations, resulting in a loss or gain of one of the six cysteine residues (4, 8, 11, 15, 25 and 28) of the EGF-like repeats located in the extracellular domain of *NOTCH3* [4]. Any mutation within the cysteine residues (a gain or loss) leads to an odd number of cysteine residues and result in impaired dimerisation of NOTCH3 or formation of inappropriate disulphide bonds causing aberrant NOTCH3 signaling [22, 23]. As such, these three mutations (p.C597W, p.C977G and p.Y1106C) were considered to be disease-causing and associated with the pure and typical pathogenetic mechanisms of CADASIL [4, 24]. The substitution of the p.C597S has been previously identified in an Arabic family [25], while the substitution of p.C977S has been reported in a Chinese patient [26] with both mutations found to be associated with CADASIL pathogenesis.

We also observed two novel amino acid substitutions (p.D139V and p.R607H) not directly involving cysteine residues, predicted to be possibly damaging and benign, respectively. As discussed by Roy et al. [17], there is some controversy over the classification non-cysteine residue altering variants and their significance to CADASIL. Several *NOTCH3* alterations that do not affect cysteine residues have been reported in families with CADASIL, which may involve other disruptions to protein function, though these may result in changes that effectively change cysteine residue availability [27–32]. It is worth noting that the predicted score for p.D139V by the SIFT programme (0.06) was more deleterious than the known pathogenic mutation p.Y465C, with a score of 0.08, technically considered to be benign. A SIFT score from 0 to 0.05 indicates that the amino acid change has a damaging effect. Further investigation of this mutation is warranted to determine the effect of these non-cysteine affecting changes on NOTCH3 function as well as on mediating signal transduction for vascular development and inducing the pathology of CADASIL. This provides new insights into the diagnosis of and pathomechanisms causing CADASIL.

The last novel synonymous variant we identified (p.E813E) was predicted by the MutationTaster programme to cause the gain of an RNA splicing donor site. This gain may result in altered protein function and therefore, despite being silent, this variant could be a real mutation causing CADASIL. Direct functional evaluation of NOTCH3 in this patient is needed to confirm this hypothesis, but such studies were not able to be performed at this time.

The in silico analysis tools to analyse the detected variants also revealed some interesting potential ramifications of the previously identified p.Y465C mutation. In 2003, Razvi et al. described this amino acid substitution as a mutation causing CADASIL [11]. In contrast, during our analysis, the computational tools predicted this amino acid change as tolerated or benign. The PhyloP score of 0.0272 and SIFT score of 0.08 (damaging score <0.05) suggest this amino acid is not conserved. The mutation is a classical CADASIL mutation; however, as stated by Joutel et al. “mutations can be unambiguously classified as pathogenic when they lead to an uneven number of cysteine residues in one of the 34 EGFR domains constituting the extracellular domain of the receptor” [24]. This discrepancy between evolutionary conservation and functional correlation models suggests caution when using functional prediction software in assigning a role to missense mutations involving cysteine residues in *NOTCH3*. The in vivo effect of amino acid substitutions should be the final arbiter for precisely describing their role in causing CADASIL, but as such tests are laborious to undertake, they are rarely performed for diagnoses. Careful consideration of the symptomatic profile may be useful in such cases and in the future when sufficient mutation data has accumulated offering clinicians more precision in ascribing the functional role of mutations in CADASIL.

Interestingly, in this study, patient C-36 demonstrated compound heterozygosity for two missense mutations in the *CACNA1A* and *SCN1A* genes (not normal target genes for CADASIL screening) [Table 1]. Mutations within these two ion channel genes are associated with various autosomal dominant disorders: hemiplegic migraine, episodic ataxia type 2, spinocerebellar ataxia type 6 and epilepsy with previously reported overlapping symptoms among these disorders [33–35]. It is worth noting that the p.E1297D mutation in *SCN1A* gene was previously reported in an Italian family with idiopathic childhood epilepsy [36]. The linkage between *CACNA1A* and *SCN1A* gene mutations and CADASIL has not previously been reported; therefore, an ongoing study in our lab will investigate the effect of these two variants/genes on CADASIL disease pathophysiology.

In terms of the clinical classification of the detected genetic variants, the full available evidence needs to be considered. Typical CADASIL mutations involve the addition or elimination of a cysteine residue in one of the 34 NOTCH3 gene epidermal growth factor (EGF)-like repeats, resulting in mismatched disulphide bridging and altered protein function, a hypothesis which has been borne out by observational and functional studies [37, 38]. Under the current American College of Medical Genetics and Genomics (ACMG) guidelines for variant classification, functional studies supporting a damaging effect for a variant on gene function constitute strong evidence for pathogenicity. Each of the cysteine altering variants also has multiple moderate and supporting lines of evidence. These include presence in a disease-associated functional domain; presence at a loci where another pathogenic mutation is known (as determined by searching HGMD, LOVD and VEP databases); absence from controls in population databases (1000 Genomes, dbSNP, ExAC); being the kind of variant (missense SNV) associated with the disease; presence in an individual with a clear phenotype; cosegregation with disease in family members (only for patients C-10 and C-44) and multiple in silico analyses predicting pathogenicity. This combination of evidence is sufficient to characterise them as pathogenic or disease-causing mutations according to the ACMG guidelines [39].

For the non-cysteine altering *NOTCH3* variants, there is less information available. Family segregation analysis and clinical information were not available for patients C-11 (p.D39V) or C-15 (p. R607H). Despite being novel amino acid changing variants in loci where disease-causing mutations are known to exist and/or functional domains, there is insufficient strength of evidence to classify either variant as pathogenic or likely pathogenic. Additionally, both these patients had complex phenotypes that do not precisely map to CADASIL, and share features of episodic ataxia or familial hemiplegic migraine, indicating a potential overlapping pathophysiology or comorbidities with these disorders. Thus, these variants should be classified as variants of uncertain significance (VOUS) according to the ACMG guidelines. Patient C-24 with the synonymous variant (p.E318E) had family history indicative of CADASIL, but no other supporting evidence, though neither does the variant have any criteria for being classified as benign. This variant has also been classified as a VOUS.

Patient C-36, who bears variants in both *CACNA1A* and *SCN1A* also, had no family members available for further investigation. Neither variant has sufficient evidence to indicate direct pathogenicity, despite being in regions of these genes known to harbour disease-causing mutations. Additionally, their presence in a gene which causes symptoms overlapping with CADASIL indicates a possible complex pathophysiology that requires more research. Hence, these variants have been classified as VOUS [39].

Finally, nine rare variants were identified in nine patients with no other causative mutation in *NOTCH3* [Table 3]. Of these, three amino acid changing variants (p.S497L, p.A1020P and p.H1133Q) were recently reported by Abramycheva et al. [15] as normal polymorphisms in Russian CADASIL patients. However, in this study, patient C-3 was found to carry non-cysteine *NOTCH3* gene variants (p.S496L and p.A1020P). As yet, a comparison of the effect of these two non-cysteine variants on the pathogenic mechanisms of CADASIL or CADASIL-like phenotype [16] to a single non-cysteine variant on disease pathogenesis has not been functionally tested.

We have identified classical CADASIL-causing mutations as well as a number of amino acid changing variants that have uncertain causative effects on this disease. The study of a larger population cohort of cases including symptomatic detail will likely provide more clinical and molecular information about their impact as well as the potential effect of any rare SNPs. Most interestingly, our results indicate that there may be other CADASIL gene/genes yet to be identified for inclusion in future diagnostic arrays.

## Conclusions

NGS technologies provide an effective method for CADASIL and related disease diagnosis. Sequencing large but targeted regions of interest of pooled DNA from multiple samples is a promising tool for the discovery of both known and novel variants associated with disease. Compared with traditional SS, the NGS platform provides increased accuracy along with reduced time and assay costs necessary to perform routine genetic diagnosis of CADASIL in ethnically heterogeneous populations, putting such testing within reach of more patients.

## Materials and methods

### Patients

Forty-four patients with a suspected clinical diagnosis of CADASIL were re-screened using the NGS approach. Patients referred to the Genomics Research Centre (GRC) diagnostic laboratory for CADASIL molecular testing through neurologists from Australia and New Zealand and showed no mutations when using SS in our standard exon sequencing (3 and 4) at the first stage and (2, 11, 18 and 19) second stage [17]. Re-sequencing of the 44 patients was based on the clinical information had provided (i.e. positive skin biopsy results for CADASIL or white matter changes in their MRI) indicating that CADASIL-causing mutations may be present.

### Molecular analysis

#### Ion AmpliSeq custom panel design

The AmpliSeq design target used in this report comprised the coding exons, exon/intron junctions and UTR regions of the *NOTCH3* gene. The Ampliseq automated primer design tool (http://www.ampliseq.com) was used to design primers covering 92.79 % of the desired target area (8071 bp) aligned to the reference human genome (hg 19). The missing regions include a 175 bp region in exon 1 (position 15311617-15311792 on chromosome 19) and a 407 bp region in exon 24 (position 15288427-15288834 on chromosome 19). The remainder of the 33 exons in the *NOTCH3* gene were included at 100 % coverage.

#### Library preparation

Genomic DNA was previously purified from peripheral blood samples using standard extraction conditions using Qiagen QIAamp DNA Blood Midi Kits as recommended by the manufacturer. The Qubit dsDNA High Sensitivity (HS) Assay Kit (Thermo Fisher Scientific, Scoresby, Victoria, Australia) was used to ensure accuracy of DNA concentration input (10 ng/μL) to NGS library construction.

Library preparation was performed using the Ion AmpliSeq library kit 2.0 (Thermo Fisher Scientific, Scoresby, Victoria, Australia) according to the standard protocol (Cat. no. 4480441, Rev. 4.0). Briefly, for the multiplex PCR amplification, 10 ng of each genomic DNA sample was amplified using the optimised modification method generated in our laboratory allowing each primer pool to be amplified as a 5-μL reaction, rather than a 20-μL reaction (protocol is available upon request). This was performed using 1 μL of 5× Ion AmpliSeq HiFi Master Mix, 2.5 μL of 2× AmpliSeq Custom primer pool, 0.5 μL nuclease-free water and 1 μL (10 ng/μL) of DNA. The reaction mix was heated for 2 min at 99 °C for enzyme activation, followed by 18 two-step cycles of 99 °C for 15 s and 60 °C for 4 min, ending with a holding period at 10 °C.

After cycling, the two 5 μL/reaction pools for each sample were combined into a single well with a total volume 10 μL. The pooled amplified samples were partially digested using 1 μL FuPa enzyme per sample at 50 °C for 10 min and 55 °C for 10 min followed by enzyme inactivation at 60 °C for 20 min. To enable multiple sample libraries to be loaded per chip, 2 μL of a unique diluted Barcode Adapter mix including Ion Xpress Barcode (numbered 1-16) and Ion P1 Adaptor at standard volumes was ligated to the end of the digested amplicons using 1 μL DNA ligase for 30 min at 22 °C followed by ligase inactivation for 10 min at 72 °C. The resulting unamplified adaptor-ligated libraries were purified using the 22.5 μL Agencourt AMPure XP system (Beckman Coulter, Brea, CA, USA) followed by addition of 75 μL freshly prepared 70 % ethanol to each library.

After purification, the amplicon libraries were further amplified to enrich material for accurate quantification using 25 μL Platinum PCR SuperMix High Fidelity and 1 μL of library Amplification Primer Mix (Ion AmpliSeq library kit 2.0, Thermo Fisher Scientific, Scoresby, Victoria, Australia), at 98 °C for 2 min followed by five two-step cycles of 98 °C for 15 s and 60 °C for 1 min. The amplified amplicon libraries were then purified using 12.5 μL Agencourt AMPure XP Reagent followed by a second purification step with 30 μL AMPure XP and 75 μL of freshly prepared 70 % ethanol added to each library. The concentration and size of amplicons was then determined using an Agilent BioAnalyzer DNA High-Sensitivity chip (Agilent Technologies, Santa Clara, CA, USA), according to manufacturers’ instructions. After quantification, each library was diluted to a concentration of ~10 pM prior to template preparation. Subsequently, libraries (*n* = 16) were pooled in equimolar amounts prior to further processing.

#### Template preparation (emulsion PCR) and sequencing

Emulsion PCR, emulsion breaking and enrichment (template preparation) were performed using the Ion PGM OT2 200 Template Kit (Thermo Fisher Scientific, Scoresby, Victoria, Australia), according to the manufacturers’ instructions (part no. 4480974 Rev. 4.0).

After preparation of the ISPs, sequencing was performed with an Ion Torrent Personal Genome Machine (PGM) system using Ion Sequencing 200 Kit V2 and an Ion 316 Chip (Thermo Fisher Scientific, Scoresby, Victoria, Australia) according to the manufacturers’ procedures (Cat. no.4482006 Rev.1.0).

#### Bioinformatic analyses

The Ion Torrent PGM sequence data was mapped to the complete human genome (hg19) by the Ion Torrent Suite software and Torrent Server along with Torrent Mapping Alignment Program optimised to Ion Torrent data. The bam format file generated by Torrent Suite was uploaded and visualised for human examination using Integrative Genomics Viewer (IGV) 2.3 software [12]. The Ion Reporter software 4.0 (Thermo Fisher Scientific, Scoresby, Victoria, Australia) was used to analyse data from Torrent PGM. The software identifies variants and performs automated annotation on Ion PGM data. Variants were classified into simple categories, summarised into a report which included links to appropriate databases for known variants.

DNA and protein sequences from NGS and SS were compared with the NCBI reference sequences [13] and the UCSC genome browser [40]. All rs ID numbers, locations, allele frequencies and genotypes for all variants were determined based on SNPs reported in the dbSNP database [41] and further analysed in the 1000 Genomes data set. To predict the effect of non-synonymous single nucleotide substitutions on protein structure, function or phenotype, we used the wANNOVAR programme [42, 43] which included the use of five functional prediction software programmes for non-synonymous variants (PhyloP [44], SIFT [45], PolyPhen2 [46], MutationTaster [47] and GERP++ [48]). In silico prediction programmes including AGVGD [49] and PhD-SNP [50] were also used to predict causative variants. For synonymous variants and variants in non-coding regions, the MutationTaster [47] software alone was used. All variants detected were examined for associated information in the public databases (at a minimum, dbSNP, OMIM, LOVD, 1000 Genomes and HGMD) and in the published literature.

#### Sanger sequencing (SS)

All detected novel mutations by NGS were further investigated by SS. Molecular analysis of the *NOTCH3* gene was performed according to a previously described protocol [17]. Briefly, genomic DNA was extracted using Qiagen QIAamp DNA Blood Midi kits. DNA was amplified by PCR to screen the exons containing novel mutations and was performed with the primers shown in Additional file 3: Table S3 online. PCR amplification for all exons were conducted as previously described [17], and cycling protocols is available for all exons upon request. PCR products were purified using Affymetrix ExoSap-IT reagent (ExoSap-IT, USB Corporation, Staufen, Germany) and directly sequenced for both sense and antisense strands using Big Dye Terminator V3.1 (Applied Biosystems, Foster City, CA, USA) on an ABI 3500 Genetic Analyser (Applied Biosystems) according to established procedures. Sequences were analysed with Chromas 2.33 software (Technelysium, Brisbane, Queensland, Australia).

## Additional files

Additional file 1: Table S1. Variants detected in *NOTCH3* gene by NGS in all 40 patients the transcript RefSeq NM_000435.2. (DOC 75 kb)
Additional file 2: Table S2. Overview of clinical data for patients tested using the NGS panel with a clinical diagnosis of CADASIL. (DOC 59 kb)
Additional file 3: Table S3. List of primers used for PCR amplification of exons with novel *NOTCH3* mutations and variants detected by NGS. (DOC 28 kb)

## Abbreviations

CADASIL: Cerebral autosomal dominant arteriopathy with subcortical infarcts and leukoencephalopathy
DNA: Deoxyribonucleic acid
EGF: Epidermal growth factor
GERP: Genomic evolutionary rate profiling
GOM: Granular osmiophilic material
GRC: The Genomics Research Centre
IGV: Integrative Genomics Viewer
NATA: National Association of Testing Authorities, Australia
NCBI: National Centre for Biotechnology Information
NGS: Next-generation sequencing
*NOTCH3*: Notch, Drosophila, Homolog of, 3
PCR: Polymerase chain reaction
PGM: Personal Genome Machine
SNPs: Single nucleotide polymorphisms
UTR: Untranslated region

## References

[1] Chabriat H (1995). Clinical spectrum of CADASIL: a study of 7 families. Cerebral autosomal dominant arteriopathy with subcortical infarcts and leukoencephalopathy. Lancet.

[2] Ruchoux MM (1994). Presence of ultrastructural arterial lesions in muscle and skin vessels of patients with CADASIL. Stroke.

[3] Chabriat H (1995). Autosomal dominant migraine with MRI white-matter abnormalities mapping to the CADASIL locus. Neurology.

[4] Joutel A (1996). Notch3 mutations in CADASIL, a hereditary adult-onset condition causing stroke and dementia. Nature.

[5] Thomsen LL, Olesen J, Russell MB (2003). Increased risk of migraine with typical aura in probands with familial hemiplegic migraine and their relatives. Eur J Neurol.

[6] Vedeler C, Bindoff L (2011). A family with atypical CADASIL. J Neurol.

[7] Maksemous N (2016). Next-generation sequencing identifies novel CACNA1A gene mutations in episodic ataxia type 2. Mol Genet Genomic Med.

[8] Dichgans M (2000). Small in-frame deletions and missense mutations in CADASIL: 3D models predict misfolding of Notch3 EGF-like repeat domains. Eur J Hum Genet.

[9] Oliveri RL (2001). A novel mutation in the Notch3 gene in an Italian family with cerebral autosomal dominant arteriopathy with subcortical infarcts and leukoencephalopathy: genetic and magnetic resonance spectroscopic findings. Arch Neurol.

[10] Tang SC (2005). Arg332Cys mutation of NOTCH3 gene in the first known Taiwanese family with cerebral autosomal dominant arteriopathy with subcortical infarcts and leukoencephalopathy. J Neurol Sci.

[11] Razvi SS (2003). Diagnostic strategies in CADASIL. Neurology.

[12] Thorvaldsdottir H, Robinson JT, Mesirov JP (2013). Integrative Genomics Viewer (IGV): high-performance genomics data visualization and exploration. Brief Bioinform.

[13] Pruitt KD (2012). NCBI Reference Sequences (RefSeq): current status, new features and genome annotation policy. Nucleic Acids Res.

[14] Markus HS (2002). Diagnostic strategies in CADASIL. Neurology.

[15] Abramycheva N (2015). New mutations in the Notch3 gene in patients with cerebral autosomal dominant arteriopathy with subcortical infarcts and leucoencephalopathy (CADASIL). J Neurol Sci.

[16] He D (2016). The comparisons of phenotype and genotype between CADASIL and CADASIL-like patients and population-specific evaluation of CADASIL scale in China. J Headache Pain.

[17] Roy B (2012). Two novel mutations and a previously unreported intronic polymorphism in the NOTCH3 gene. Mutat Res.

[18] Bianchi S (2015). CADASIL in central Italy: a retrospective clinical and genetic study in 229 patients. J Neurol.

[19] Fernandez A (2015). A next-generation sequencing of the NOTCH3 and HTRA1 Genes in CADASIL Patients. J Mol Neurosci.

[20] Adib-Samii P (2010). Clinical spectrum of CADASIL and the effect of cardiovascular risk factors on phenotype: study in 200 consecutively recruited individuals. Stroke.

[21] Kim YE (2014). Spectrum of NOTCH3 mutations in Korean patients with clinically suspicious cerebral autosomal dominant arteriopathy with subcortical infarcts and leukoencephalopathy. Neurobiol Aging.

[22] Joutel A (2004). Pathogenic mutations associated with cerebral autosomal dominant arteriopathy with subcortical infarcts and leukoencephalopathy differently affect Jagged1 binding and Notch3 activity via the RBP/JK signaling Pathway. Am J Hum Genet.

[23] Wang W (2002). Notch3 signaling in vascular smooth muscle cells induces c-FLIP expression via ERK/MAPK activation. Resistance to Fas ligand-induced apoptosis. J Biol Chem.

[24] Joutel A (2013). Loss-of-function mutation in the NOTCH3 gene: simply a polymorphism?. Hum Mutat.

[25] Bohlega S (2011). Novel mutation of the notch3 gene in arabic family with CADASIL. Neurol Int.

[26] Lee YC (2006). Cerebral autosomal dominant arteriopathy with subcortical infarcts and leukoencephalopathy: two novel mutations in the NOTCH3 gene in Chinese. J Neurol Sci.

[27] Brass SD (2009). Case records of the Massachusetts General Hospital. Case 12-2009. A 46-year-old man with migraine, aphasia, and hemiparesis and similarly affected family members. N Engl J Med.

[28] Kim Y (2006). Characteristics of CADASIL in Korea: a novel cysteine-sparing Notch3 mutation. Neurology.

[29] Mazzei R (2004). A novel Notch3 gene mutation not involving a cysteine residue in an Italian family with CADASIL. Neurology.

[30] Santa Y (2003). Genetic, clinical and pathological studies of CADASIL in Japan: a partial contribution of Notch3 mutations and implications of smooth muscle cell degeneration for the pathogenesis. J Neurol Sci.

[31] Scheid R (2008). Cysteine-sparing notch3 mutations: cadasil or cadasil variants?. Neurology.

[32] Uchino M (2002). Cerebral autosomal dominant arteriopathy with subcortical infarcts and leukoencephalopathy (CADASIL) and CADASIL-like disorders in Japan. Ann N Y Acad Sci.

[33] Herman-Bert A (2000). Mapping of spinocerebellar ataxia 13 to chromosome 19q13.3-q13.4 in a family with autosomal dominant cerebellar ataxia and mental retardation. Am J Hum Genet.

[34] Ophoff RA (1996). Familial hemiplegic migraine and episodic ataxia type-2 are caused by mutations in the Ca2+ channel gene CACNL1A4. Cell.

[35] Wallace RH (2001). Neuronal sodium-channel alpha1-subunit mutations in generalized epilepsy with febrile seizures plus. Am J Hum Genet.

[36] Orrico A (2009). Mutational analysis of the SCN1A, SCN1B and GABRG2 genes in 150 Italian patients with idiopathic childhood epilepsies. Clin Genet.

[37] Arboleda-Velasquez JF (2005). CADASIL mutations impair Notch3 glycosylation by Fringe. Hum Mol Genet.

[38] Opherk C (2009). CADASIL mutations enhance spontaneous multimerization of NOTCH3. Hum Mol Genet.

[39] Richards S (2015). Standards and guidelines for the interpretation of sequence variants: a joint consensus recommendation of the American College of Medical Genetics and Genomics and the Association for Molecular Pathology. Genet Med.

[40] Dreszer TR (2012). The UCSC Genome Browser database: extensions and updates 2011. Nucleic Acids Res.

[41] Sherry ST (2001). dbSNP: the NCBI database of genetic variation. Nucleic Acids Res.

[42] Chang X, Wang K (2012). wANNOVAR: annotating genetic variants for personal genomes via the web. J Med Genet.

[43] Wang K, Li M, Hakonarson H (2010). ANNOVAR: functional annotation of genetic variants from high-throughput sequencing data. Nucleic Acids Res.

[44] Pollard KS (2010). Detection of nonneutral substitution rates on mammalian phylogenies. Genome Res.

[45] Ng PC, Henikoff S (2001). Predicting deleterious amino acid substitutions. Genome Res.

[46] Adzhubei IA (2010). A method and server for predicting damaging missense mutations. Nat Methods.

[47] Schwarz JM (2010). MutationTaster evaluates disease-causing potential of sequence alterations. Nat Methods.

[48] Davydov EV (2010). Identifying a high fraction of the human genome to be under selective constraint using GERP++. PLoS Comput Biol.

[49] Tavtigian SV (2006). Comprehensive statistical study of 452 BRCA1 missense substitutions with classification of eight recurrent substitutions as neutral. J Med Genet.

[50] Capriotti E, Calabrese R, Casadio R (2006). Predicting the insurgence of human genetic diseases associated to single point protein mutations with support vector machines and evolutionary information. Bioinformatics.

