# Supplementary material for: Targeted next generation sequencing identifies novel NOTCH3 gene mutations in CADASIL diagnostics patients
Source: Hum Genomics. 2016 Nov 24;10:38. doi: 10.1186/s40246-016-0093-z (PMC5122195; doi:10.1186/s40246-016-0093-z)
Supplement: Additional file 1: Table S1. — Variants detected in NOTCH3 gene by NGS in all 40 patients the transcript RefSeq NM_000435.2. (DOC 75 kb) [file 40246_2016_93_MOESM1_ESM.doc]

Additional file 1: Table S1: Variants detected in *NOTCH3* gene by NGS in all forty patients the transcript RefSeq NM_000435.2

| Chr | Position^1^ | dbSNP | MAF | Ref (Rev) | Genotype | Location | Function | Protein Change |
| --- | --- | --- | --- | --- | --- | --- | --- | --- |
| 19 | 15270583 | rs1044123 | G=0.375 | G | G,A | utr_3 | - | - |
| 19 | 15270636 | rs12082 | T=0.039 | C | C,T | utr_3 | - | - |
| 19 | 15270665 | rs1044116 | T=0.375 | C | C,T | utr_3 | - | - |
| 19 | 15270805 | rs1044055 | A=0.376 | C | C,A | utr_3 | - | - |
| 19 | 15271135 | rs7247906 | G=0.132 | T | G,G | utr_3 | - | - |
| 19 | 15271771 | rs1044009 | A=0.369 | G | G,A | exonic | [missense] | p.Ala2223Val |
| 19 | 15272001 | rs1044008 | T=0.023 | C | C,T | exonic | [synonymous] | WT |
| 19 | 15273248 | rs4809029 | C=0.132 | A | C,C | intronic | - | - |
| 19 | 15273381 | rs4809030 | G=0.134 | A | G,G | intronic | - | - |
| 19 | 15276739 | rs16980398 | G=0.091 | A | A,G | exonic | [synonymous] | WT |
| 19 | 15278057 | rs1548555 | G=0.132 | A | G,G | intronic | - | - |
| 19 | 15281342 | rs149222385 | C=0.001 | T | T,C | exonic | [synonymous] | WT |
| 19 | 15281386 | rs2074620 | C=0.437 | C | A,A | intronic | - | - |
| 19 | 15281459 | rs56277836 | G=0.056 | C | C,G | intronic | - | - |
| 19 | 15285052 | rs1044006 | C=0.133 | T | C,C | exonic | [synonymous] | WT |
| 19 | 15289613 | rs11670823 | T=0.464 | A | A,T | intronic | - | - |
| 19 | 15289665 | - | - | C | C,A | exonic | [missense] | p.Gly1269Val |
| 19 | 15290007 | rs10408676 | T=0.067 | C | C,T | exonic | [missense] | p.Val1183Met |
| 19 | 15290236 | rs112197217 | T=0.006 | G | G,T | exonic | [missense] | p.His1133Gln |
| 19 | 15290893 | - | - | T | T,C | exonic | [missense] | P.Tyr1106Cys* |
| 19 | 15291576 | rs35769976 | G=0.084 | C | C,G | exonic | [missense] | p.Ala1020Pro |
| 19 | 15291837 | - | - | A | A,C | exonic | [missense] | p.Cys977Gly* |
| 19 | 15292366 | rs11669982 | T=0.233 | C | C,T | intronic | - | - |
| 19 | 15292437 | rs1043997 | C=0.233 | T | T,C | exonic | [synonymous] | WT |
| 19 | 15295134 | rs1043996 | A=0.462 | G | G,A | exonic | [synonymous] | WT |
| 19 | 15295233 | - | - | C | C,T | exonic | [synonymous] | WT* |
| 19 | 15296513 | rs147014533 | T=0.006 | C | C,T | intronic | - | - |
| 19 | 15297936 | - | - | C | C,T | exonic | [missense] | p.Arg607His* |
| 19 | 15297965 | - | - | G | G,C | exonic | [missense] | p.Cys597Trp* |
| 19 | 15298031 | rs79926127 | T=0.005 | C | C,T | exonic | [synonymous] | WT |
| 19 | 15299048 | rs114207045 | A=0.006 | G | G,A | exonic | [missense] | p.Ser497Leu |
| 19 | 15299051 | rs11670799 | A=0.006 | G | G,A | exonic | [missense] | p.Pro496Leu |
| 19 | 15299144 | - | - | T | T,C | exonic | [missense] | p.Tyr465Cys** |
| 19 | 15300136 | rs61749020 | G=0.014 | A | A,G | exonic | [synonymous] | WT |
| 19 | 15302277 | - | - | G | G,A | exonic | [missense] | p.Arg332Cys** |
| 19 | 15302790 | rs114457076 | A=0.002 | G | G,A | exonic | [synonymous] | WT |
| 19 | 15302844 | rs1043994 | C=0.144 | T | C,C | exonic | [synonymous] | WT |
| 19 | 15302903 | - | - | A | A,G | exonic | [missense] | p.Cys183Arg** |
| 19 | 15303034 | - | - | T | T,A | exonic | [missense] | p.Asp139Val* |
| 19 | 15303225 | rs3815188 | A=0.238 | G | G,A | exonic | [synonymous] | WT |
| 19 | 15308287 | rs188132716 | C=0.006 | G | G,C | intronic | - | - |
| 19 | 15308288 | rs202151374 | T=0.003 | G | G,T | intronic | - | - |

* Novel mutations and variants identified- **Known mutations detected. ^1^Position based on hg19 release
