# Supplementary material for: Targeted next generation sequencing identifies novel NOTCH3 gene mutations in CADASIL diagnostics patients
Source: Hum Genomics. 2016 Nov 24;10:38. doi: 10.1186/s40246-016-0093-z (PMC5122195; doi:10.1186/s40246-016-0093-z)
Supplement: Additional file 2: Table S2. — Overview of clinical data for patients tested using the NGS panel with a clinical diagnosis of CADASIL. (DOC 59 kb) [file 40246_2016_93_MOESM2_ESM.doc]

**Additional file 2:** **Table S2**. Overview of clinical data for patients tested using the NGS panel with a clinical diagnosis of CADASIL.

| Patient ID | Age | Gender | Clinical Phenotype |
| --- | --- | --- | --- |
| C-1 | 37 | F | Long history of migraine, headaches and cognitive symptoms. white matter changes on MRI |
| C-2 | 17 | F | Vestibular migraines, dizziness, crawling sensation on skin, nausea, imbalance most days for several hours. Phx amikacin/gentamycin caused deafness 2004 |
| C-3 | 47 | M | Positive skin biopsy for CADASIL on EM, two daughters with intellectual handicap |
| C-4 | 67 | M | Overlapping symptoms between Familial hemiplegic migraine and CADASIL, Diagnosis was FHM but neurologist requested both Familial Hemiplegic Migraine (CACNA1A, ATP1A2 and SCN1A) and NOTCH3 gene testing |
| C-5 | 58 | F | Unusual reaction to a psychotropic agent followed by a period of confusion, resembling a rapid dementia, however, patient recovered fully. MRI scan showed extensive white matter disease resembling CADASIL. Skin biopsy performed and considered positive. Patient does not suffer from migraine, has had no strokes and does not appear to be dementing. |
| C-6 | 51 | F | Positive skin biopsy for CADASIL, migraines, TIA,s, CVA family history (see pedigree) |
| C-7 | 65 | F | Positive skin biopsy, dementia and positive MRI |
| C-8 | 37 | F | Equivocal skin biopsy for CADASIL, headaches-cause unclear, Coronary artery spasm, |
| C-9 | 62 | F | positive skin biopsy for CADASIL |
| C-10 | 74 | F | Family history of cadasil and positive skin biopsy in patient and two first degree relatives |
| C-11 | 42 | F | Small vessel changes, ataxia type 1 |
| C-12 | 56 | F | Two cerebrovascular events, MRI shows subcortical lecoencephalopathy and positive skin biopsy |
| C-13 | 56 | M | CADASIL-Like illness, white matter abnormality, migraine, seizures and probable cognitive change. Skin biopsy change |
| C-14 | 33 | M | Father has CADASIL, positive skin biopsy and familial mutation found in exon 9. |
| C-15 | 52 | F | Overlapping symptoms between Familial Hemiplegic Migraine and CADASIL, Diagnosis was FHM but neurologist requested both Familial Hemiplegic Migraine (CACNA1A, ATP1A2 and SCN1A) and NOTCH3 gene testing |
| C-16 | 65 | F | Has attacks of speech disturbance in which speech is disrupted for several days but comprehension unaffected. Suffers from migraine. EEG, MRI and CT scan showed no abnormality. |
| C-17 | 55 | M | Clinical information not available |
| C-18 | 21 | F | Recurrent hemiparesis |
| C-19 | 46 | F | Headaches, weakness, MRI white matter changes |
| C-20 | 54 | F | Recurrent migrainous events, TIAs and strokes with subcortical white matter ischaemic changes. Warfarin has improved frequency of her symptoms. Family history of cardiovascular events. Positive skin biopsy. |
| C-21 | 54 | F | TIAs improved by anticoagulants ceased. MRI subcortical changes, family history with two first degree relatives |
| C-22 | 52 | F | Clinical information not available |
| C-23 | 56 | M | Several stokes, speech disturbance, white matter changes on MRI |
| C-24 | 54 | M | Subcortical infarct type changes on MRI, atypical migraine, mother CVA aged 62, maternal uncle dementia aged 60, another uncle CVA |
| C-25 | 51 | M | Hemiparetic migraine, CVAs, MRI with matter changes, negative skin biopsy |
| C-26 | 38 | M | MRI changes, frontal occipital periventricular, headaches, lethargy memory loss, |
| C-27 | 46 | F | Monophasic demyelinating disease, subcortical stroke |
| C-28 | 60 | M | Life-long history of headaches, TIAs or seizures with transient loss of motor function. CT scan with periventricular white matter changes. Mother died of CVA aged 50's. Family history of migraine |
| C-29 | 66 | F | Positive skin biopsy |
| C-30 | 17 | M | Seizures, behavioural disorder, MRI shows white matter hyper intensity both cerebral hemispheres, including temporal polar lobes |
| C-31 | 65 | F | CNS demyelination/ stroke |
| C-32 | 39 | F | Clinical information not available |
| C-33 | 61 | F | Clinical diagnosis of CADASIL |
| C-34 | 59 | F | Cognitive decline, MRI shows extensive confluent white matter change and cortical atrophy. Family History of stroke, dementia and migraine. |
| C-35 | 43 | F | Cognitive impairment, MRI appearance consistent with CADASIL |
| C-36 | 60 | M | Clinical information not available |
| C-37 | 52 | F | Negative skin biopsy |
| C-38 | 58 | F | Progressive neurological disorder, MELAS, CADASIL , Mitochondrial OR Metabolic Syndrome |
| C-39 | 55 | M | Clinical information not available |
| C-40 | 65 | F | Clinical information not available |
| C-41 | 60 | F | Dementia, seizures, extensive white matter changes in typical CADASIL distribution, identified twin sibling has confusion and cognitive decline over past 2 years, sister’s MRI shows non-specific white matter changes |
| C-42 | 47 | F | CADASIL diagnosis and MRI confirmed. |
| C-43 | 39 | M | Family history, paternal grandfather, father both with early strokes. Uncle and brother both have genetic confirmation of CADASIL. |
| C-44 | 52 | F | Family history of CADASIL, positive skin biopsy in patient. |
