# Supplementary material for: Targeted next generation sequencing identifies novel NOTCH3 gene mutations in CADASIL diagnostics patients
Source: Hum Genomics. 2016 Nov 24;10:38. doi: 10.1186/s40246-016-0093-z (PMC5122195; doi:10.1186/s40246-016-0093-z)
Supplement: Additional file 3: Table S3. — List of primers used for PCR amplification of exons with novel NOTCH3 mutations and variants detected by NGS. (DOC 28 kb) [file 40246_2016_93_MOESM3_ESM.doc]

Additional file 3: Table S3: List of primers used for PCR amplification of exons with novel *NOTCH3* mutations and variants detected by NGS.

| Exon | Sense primer (5’-3’) | Antisense primer(5’-3’) |
| --- | --- | --- |
| 4 | TAGTCGGGGGTGTGGTCAGT | TCAAACCCTAGCAGGGAA |
| 11 | ATTGGTCCGAGGCCTCACTT | CCATTCCCAACCCCTCTGTG |
| 16 | AATGCCCAGACACGAATGA | CGGGCCTCAGTTTCCATATAA |
| 18 | GATCCTCCCTCCCACTCCTT | GGGGAAGCACTCAGAGTCAG |
| 20 | TGTGTGATGGAGGCAGAAG | ATACCCATACCAAGCCACAC |
| 23 | ATTGTGGCTGATCTACATGCTC | CCACATCCTCCTCCTAGACG |
